# Deep‐Learning Algorithm Diagnostic Support for Usual Interstitial Pneumonia Pattern Recognition in Fibrotic Interstitial Lung Disease

**DOI:** 10.1002/resp.70246

**Published:** 2026-04-01

**Authors:** Caitlin C. Fermoyle, John A. Mackintosh, Vidya Navaratnam, Samantha J. Ellis, Wendy A. Cooper, Nicole S. L. Goh, Yuben Moodley, Paul N. Reynolds, Christopher J. Zappala, Peter Hopkins, Ian N. Glaspole, Tamera J. Corte, Simon L. F. Walsh, the SOFIA Project Consortium, Daniele Accornero, Aditya Agrawal, Isil Kibar Akilli, Omer Alamoudi, Maria Laura Alberti, Rasoul Aliannejad, Hamdan Aljahdali, Gina Amanda, Reut Anconina, Julio Daniel Antuni, Giuseppe Aquaro, Juan Arenas‐Jimenez, Bassey Asuquo, Iain Au‐Yong, Sergey Avdeev, Maurizio Balbi, Bruno Baldi, Elisabetta Balestro, Andrea Yu‐Lin Ban, Fotini Bardaka, Nicola Boscolo Bariga, Dhiraj Baruah, Ionela Belaconi, Elisabeth Bendstrup, David Bennett, Hans‐Christian Blum, Marialuisa Bocchino, Samuel De Bontridder, Andrea Borghesi, Demosthenes Bouros, Gracijela Bozovic, Pierre‐Yves Brillet, Marsel Broqi, John Bruzzi, Suryakala Buddha, Ivette Buendia‐Roldan, Carolina Cabo, Maria del Carmen Venero Caceres, Cristina Calandra, Roberto Calandriello, Diana Calaras, Jack Callum, Paula Campos, Roberto Carbone, Fabian Caro, Andre Carvalho, Marcelo Figueroa Casas, Eva Castaner, Jesus Javier Diaz Castanon, Cecilio Ceballos, Lorenzo Cereser, Veli Cetinsu, Gin Tsen Chai, Sachin Chaudhary, Nazia Chaudhuri, Chih‐Yu Chen, Patrick Alain Chui Wan Cheong, Giuseppe Cicchetti, Annemilia del Ciello, Alessandro Balbiano di Colcavagno, Sahary Conde, Pietro Costantini, Vincent Cottin, Davide Coviello, Diletta Cozzi, Dumitru Cravcenco, Giuseppe Cutaia, Rosa D'Abronzo, Gabriele D'Andrea, Marie‐Pierre Debray, Perla Delgado, Diemen Delgado‐Garcia, Alain Delobbe, Jane Dematte, Devesh J. Dhasmana, Sahajal Dhooria, Fotios Drakopanagiotakis, Dildar Duman, Chary Duraikannu, Glenn Eiger, Karim El‐Kersh, Samantha Ellis, Juan Ignacio Enghelmayer, Rosa Estrada‐Y‐Martin, Sherene Fakhran, Alessandra Farchione, Mary Jo Farmer, Alexia Farrugia, Leandro Fassola, Paola Faverio, Federico Felder, Maria Fernandez‐Velilla, Gilbert Ferretti, Justyna Fijolek, Francesco Filippone, Kevin Flaherty, Fabio Franco, Domenico Salvatore Gagliano, Amaia Urrutia Gajate, Clara Patricia Garcia, Andrea Estrada Garrido, Adrian Gaser, Bindu George, Subha Ghosh, Eddie Gibson, Hester Gietema, Rodrigo Gil, Beatriz Liliana Gil, Ritu Gill, Georgia Gkrepi, Raul Godoy, Athena Gogali, Nicole Goh, Alejandro Gomez, Aleksandar Grgic, Julien Guiot, H Henry Guo, Amit Gupta, Richard Hammond, Simon Hart, Thomas Hartman, Michael Henry, Nik Hirani, Wan Chin Hsieh, Killian Hurley, Charlotte Hyldgaard, Daniela Buklioska Ilievska, Marta Inchausti, Yoshikazu Inoue, Dominique Israel‐Biet, Maham Jehangir, Kerri Johannson, Takeshi Johkoh, Janet Johnston, Fortunato Juarez‐Hernandez, Soma Jyothula, Yasemin Kabasakal, Meena Kalluri, Can Zafer Karaman, Peter Kardos, Sandeep Katiyar, Ravindra Kumar Kedia, Lan‐Chau Kha, Nasreen Khalil, Mohammad Ayaz Khan, Yet H. Khor, Arda Kiani, Tomoo Kishaba, Heiko Knoop, Umut Knoop, Jane Ko, Eva Kocova, Lykourgos Kolilekas, Yasuhiro Kondoh, Chi Wan Koo, Vasileios Kouranos, Martijn de Kruif, Melahat Kul, Ozlem Ozdemir Kumbasar, Ronald Kuzo, Hoi Yee Kwan, Sebastien Van Laethem, Nicholas Landini, David Lang, Anna Rita Larici, Esther Law, Roberta Eufrasia Ledda, Ivo van der Lee, Yunkai Li, Valencia Lim, Randolph Lipchik, Su Ying Low, Fabrizio Luppi, Foteini Malli, Milena Adina Man, Eliane Mancuzo, Silvina Mannarino, George Margaritopoulos, Cristina Marrocchio, Manuela Martinez‐Frances, Toshiaki Matsuda, Federico Mei, Mayra Mejia, Veronica Menardi, Mikel Mendoza, Aravind Menon, Patricia Lopez Miguel, Ruxandra‐Iulia Milos, Paul Minnis, Atsushi Miyamoto, Nesrin Mogulkoc, Maria Molina‐Molina, Michele Mondoni, Zsuzsanna Monostori, Brian Morrissey, Marta Garcia Moyano, Mathias Andreas Mueller, Suranjan Mukherjee, Carlos F. Munoz‐Nunez, Daniel Musetescu, Prasanth Nair, Anoop Nambiar, Kiran Vishnu Narayan, Hrudaya Nath, Yuichiro Nei, Alexandra Neves, Boon Hau Ng, Tilo Niemann, Luca Novelli, Lubov Novikova, Paschalis Ntolios, Hilario Nunes, Takashi Ogura, Shinichiro Ohshimo, Anastasia Oikonomou, Maria Otaola, Marieke Overbeek, Lekshmi Padmakumari, Stefano Palmucci, Vijaya Kumary Baskara Pandian, Eftsratios Panselinas, Ilias Papanikolaou, Alessio Pascheale, Shital Patil, Wagner Diniz de Paula, Michael Perch, Raoul Pereira, Olof Joakim Pettersson, Sara Piciucchi, Wojciech Piotrowski, Roberta Polverosi, Daniel Popa, Ana Sofia Porta, Marta Posada, Thomas Skovhus Prior, Ilaria Pulzato, Mosleh Al Raddadi, Pailin Ratanawatkul, Gaetano Rea, Cristina Reichner, Pilar Rivera‐Ortega, Jonathan Rodrigues, Rui Rolo, Shigeki Saito, Yoana Lazaro Salazar, Mauricio Salinas, Mayra Alexandra Samudio, Pradosh Kumar Sarangi, Sayan Sarkar, Yuki Sato, Recep Savas, Simone Scarlata, Nicola Schembri, Thies Hendrik Schroeder, Alfredo Sebastiani, Palmi Shah, Aruna Shanmuganathan, Claudio Silva, Hans Slabbynck, Philip Slocum, Annemiek Snoeckx, Eman Sobh, Chun Ian Soo, Celia Sousa, Mark Spears, Irina Strambu, Emilia Maria Swietlik, Anne‐Marie Sykes, Pablo Szwarstein, Gabriela Tabaj, Yoshinori Tanino, Kiminobu Tanizawa, Adam Domonkos Tarnoki, David Laszlo Tarnoki, Felicia Teo, Weiping Tham, Fernando Tirapegui, Ryuichi Togawa, Claudia Lucia Toma, Sara Tomassetti, Keisuke Tomii, Hiromi Tomioka, Ioannis Tomos, Abdelfattah Touman, Sergio Trujillo, Vasilios Tzilas, Argyris Tzouvelekis, Marcela Usandivaras, Clara Valsecchi, Francesco Varone, Gerson Velasquez‐Pinto, Oksana Viltsaniuk, Yagnang Vyas, Yuko Waseda, Yuranga Weerakkody, Margaret Wilsher, Wim Wuyts, Oleh Yakovenko, Esteban Zirulnik, Maurizio Zompatori

**Affiliations:** ^1^ Faculty of Medicine and Health University of Sydney Sydney Australia; ^2^ Faculty of Medicine University of Queensland Brisbane Australia; ^3^ Department of Respiratory Medicine Sir Charles Gardiner Hospital Perth Australia; ^4^ Institute for Respiratory Health University of Western Australia Perth Australia; ^5^ Department of Radiology The Alfred Hospital Melbourne Australia; ^6^ Department of Surgery, School of Translational Medicine Monash University Melbourne Australia; ^7^ Department of Tissue Pathology and Diagnostic Oncology NSW Health Pathology, Royal Prince Alfred Hospital Sydney Australia; ^8^ School of Medicine Western Sydney University Sydney Australia; ^9^ Department of Respiratory and Sleep Medicine Austin Hospital Melbourne Australia; ^10^ Institute for Breathing and Sleep Melbourne Australia; ^11^ University of Melbourne Melbourne Australia; ^12^ Royal Perth Hospital Perth Australia; ^13^ University of Adelaide Adelaide Australia; ^14^ Royal Adelaide Hospital Adelaide Australia; ^15^ Hervey Bay Hospital Urraween Queensland Australia; ^16^ University of Queensland Brisbane Australia; ^17^ School of Medicine University of Queensland Brisbane Australia; ^18^ Alfred Hospital Melbourne Australia; ^19^ Monash University Melbourne Australia; ^20^ Royal Prince Alfred Hospital Sydney Australia; ^21^ Qureight Ltd Cambridge UK; ^22^ National Heart and Lung Institute Imperial College London UK

**Keywords:** deep learning, disease progression, idiopathic pulmonary fibrosis, radiology, usual interstitial pneumonia

## Abstract

**Background and Objective:**

High resolution computed tomography (HRCT) scan diagnostic classification for usual interstitial pneumonia (UIP) plays a critical role in therapeutic decision‐making and clinical trial eligibility for interstitial lung disease (ILD) patients, but is subject to variability. A deep learning algorithm, the Systematic Objective Fibrotic Imaging Analysis Algorithm (SOFIA), has been validated to assist classification of HRCTs based on current guidelines. In this study, we evaluate the impact of SOFIA on inter‐observer agreement for UIP classification and prognostic accuracy of clinicians' assessment of ILD HRCTs.

**Methods:**

Radiologists and pulmonologists (reviewers) were invited to evaluate 203 HRCTs from a national fibrotic ILD registry, scoring each of four UIP categories (definite UIP, probable UIP, indeterminate, or alternative diagnosis). SOFIA outputs were then provided, and reviewers were able to reevaluate their scores. Changes in interobserver agreement for UIP classification and prognostic accuracy were calculated.

**Results:**

Three hundred twelve reviewers (120 radiologists, 192 pulmonologists) from 49 countries evaluated 203 HRCT scans. Following SOFIA, inter‐observer diagnostic agreement improved for definite UIP from moderate to good (ICC_pre_ = 0.54[0.50–0.60]; ICC_post_ = 0.70[0.66–0.74]), and for probable UIP from fair to moderate (ICC_pre_ = 0.30[0.27–0.35]; ICC_post_ = 0.53[0.49–0.58]). Following SOFIA, there was improved prognostic accuracy for reviewers' definite UIP, probable UIP, and indeterminate scores (significant change in c‐index), and the proportion of reviewers whose probable UIP scores were significantly predictive of transplant‐free survival increased by 42%.

**Conclusion:**

Providing SOFIA algorithm output to clinicians reviewing HRCT scans improved diagnostic agreement and prognostic accuracy for fibrotic ILD. SOFIA may be a useful automated assistive tool to support improved diagnostic consistency.

## Abstract 2

Reproducibility of radiologist classification of pulmonary fibrosis (PF) on computed tomography scans is highly variable, with implications for management and clinical trial eligibility. A deep learning algorithm was created to aid radiologists in classifying PF scans according to current guidelines. Algorithm implementation demonstrated improved agreement and accuracy among radiologists and pulmonologists.

## 1 Introduction

High resolution computed tomography (HRCT) of the chest is a central component of the diagnosis of fibrotic lung disease. An HRCT pattern of usual interstitial pneumonia (UIP) is the key to a diagnosis of idiopathic pulmonary fibrosis (IPF), and when definite or probable UIP is present, this obviates the need to perform surgical lung biopsy according to ATS/ERS/JRS/ALAT IPF Clinical Practice Guidelines [1]. Furthermore, inclusion criteria for clinical trials of antifibrotic therapy almost universally rely on the consistent application of these guideline criteria. However, HRCT interpretation, and specifically the interpretation of these guidelines, is liable to high levels of interobserver variability and poor reproducibility even among expert radiologists [2]. Such variability in diagnosis has significant implications for management decisions and clinical trial enrolment for patients with fibrotic lung disease [3, 4].

We have reported a deep learning algorithm SOFIA (Systematic Objective Fibrotic lung disease Imaging analysis Algorithm) applied to HRCT scans, which provides an automated method for UIP classification based on guideline criteria [5]. In our prior study, SOFIA provided superior accuracy for UIP classification when compared to thoracic radiologists. However, until now, SOFIA has not been integrated into clinical decision‐making as an automated assistive tool, or applied in the setting of a clinical trial.

In this study, we prospectively evaluate the utility of the SOFIA HRCT algorithm as an automated assistive tool for HRCT interpretation by clinicians (radiologists and pulmonologists) of varying levels of experience and ILD expertise.

## 2 Methods

### 2.1 Fibrotic Interstitial Lung Disease Registry Patient Population

Anonymised HRCTs were obtained from baseline imaging stored as part of the Australian IPF Registry (AIPFR). Information on the AIPFR has been published previously [5, 6, 7]. Briefly, the AIPFR was established in 2012 and collated baseline and longitudinal clinical and imaging data. Patients were included in the AIPFR if their referring physician had clinical suspicion of IPF; however, all cases were re‐evaluated by a central multidisciplinary panel according to the IPF guideline criteria [8] with these results indicating that our patient cohort was consistent with a fibrotic ILD population including both IPF and non‐IPF disease aetiologies. Cases with volumetric HRCTs were included in this study. Clinical information from the AIPFR was used to characterise the patient cohort and evaluate concordance of reviewer scores with outcomes. The use of anonymised clinical data and images for this study was approved by Sydney Local Health District ethics committee (protocol X14‐0264).

### 2.2 Participating Reviewers

Pulmonologists and radiologists who had previously consented to be contacted about future ILD studies were invited to participate by direct email. Radiologists were derived from a pre‐existing global consortium previously described [2], which included a mix of general radiologists, chest radiologists, and thoracic radiology fellows. Additionally, an advertisement for the study was posted on LinkedIn in March 2023. Participation in the study involved completing a preliminary survey and reviewing all HRCT cases. A custom‐built website was developed to enable remote case review to be completed at any point within the 6‐month study period (March to September 2023). Consent to the study was implied if the participants completed the preliminary survey, which included questions about their primary role, location, academic status (working at a university hospital or non‐university hospital), ILD expertise (ILD expert or non‐expert), and number of years of experience. Radiologists/pulmonologists were considered ILD experts if they self‐identified as ILD specialists and reported consistent participation in ILD multidisciplinary team meetings or had completed formal ILD fellowships.

### 2.3 SOFIA‐Based Image Evaluation

SOFIA (Systematic Objective Fibrotic Imaging Analysis Algorithm) is a deep convolutional neural network based on the Inception‐ResNet‐v2 architecture proposed by Szegedy, which combines Inception modules with residual connections [9]. Development and validation of the SOFIA algorithm has been published previously [5, 10]. In brief, SOFIA was trained on a database of 420,096 unique HRCT 4‐slice montages from 1157 fibrotic lung disease specific HRCTs derived from two tertiary referral centres for ILD and validated against the performance of 92 thoracic radiologists on a test cohort of 150 HRCTs from a third institution. The algorithm's input is a four‐slice montage and its output a set of continuous numbers from 0 to 1, representing a probability for each of the UIP diagnosis categories, whose sum is 1.0 (e.g., definite UIP 0.985, probable UIP: 0.011, indeterminate: 0.002, alternative diagnosis 0.002). SOFIA generates up to 500 unique montages per HRCT scan and its final prediction for a single HRCT is the average probability assigned for each diagnostic category, for these montages.

### 2.4 Study Overview

After completing the preliminary survey, participants sequentially evaluated 203 HRCT cases, assigning a likelihood score between 0 and 100% for each of the UIP categories (definite UIP, probable UIP, indeterminate, alternative diagnosis; in increments of 5% summing to 100%). For example, a reviewer could assign a score of 70% for definite UIP, 15% for probable UIP, 10% for indeterminate, and 5% for alternative diagnosis. After providing their initial scores, reviewers were shown SOFIA outputs for the four disease pattern categories and given the option to re‐score the case. In the previous example, the reviewer might revise their definite UIP score to 80% and their indeterminate score to 0%. Importantly, reviewers evaluated each patient's HRCT scan in isolation and were not provided with additional information (e.g., demographics, clinical history, lung function).

### 2.5 Statistical Analyses

Statistical analyses were performed using STATA (version 18.5, StataCorp, College Station, TX, USA). Data are given as means with standard deviations, medians with interquartile range, or as the number of patients and percentage, where appropriate.

Agreement across the full panel of reviewers was compared using intraclass correlation coefficients (ICC) with two‐way random effects and absolute agreement [11]. An ICC of 0–0.2 was considered poor, 0.21–0.4 fair, 0.41–0.6 moderate, 0.61–0.8 good, and > 0.8 very good [12]. We explored whether viewing SOFIA outputs influences inter‐observer agreement, and whether agreement differs between pulmonologists and radiologists, those with or without ILD expertise, and between those with more or less clinical expertise (quartile with the greatest years of experience vs. quartile with the least). ICCs with non‐overlapping confidence intervals were considered to be significantly different.

Given the prognostic implications of a definite or probable UIP classification, we used prognostic accuracy as a surrogate for evaluating classification accuracy of reviewers in applying HRCT guidelines to the cases. To quantify prognostic accuracy, we determined the concordance between each reviewer's scores and patient outcomes, including both transplant‐free survival (TFS) and 12‐month progression, before and after viewing SOFIA outputs. To determine concordance of each reviewer's scores with TFS, we used Cox proportional hazards modelling to calculate a hazard ratio (HR) and Harrell's C‐index for each reviewer [13]. The survival period was calculated from the date of HRCT to date of death/transplant or last date of data collection (20 October 2020). To evaluate how well each reviewer's scores predict 12‐month disease progression as a binary outcome variable, we calculated an odds ratio and then determined the concordance (C‐index) for each reviewer. A binary 12‐month progression variable was previously calculated using linear mixed effects modelling, fitted with random slopes and intercepts, to determine disease trajectory (relative change in FVC and DLCO % predicted) for each patient across the registry follow‐up period [6]. Cases with an annual rate of relative decline in FVC > 10% or DLCO ≥ 15% were considered progressive, and all others were considered stable [14]. Pre‐ to post‐ change in C‐index was compared using a Wilcoxon signed rank test, and between group differences in change scores were compared using a Mann–Whitney U (two groups) or Kruskal–Wallis test (four groups) with a significance level of *p* < 0.05.

## 3 Results

### 3.1 Fibrotic ILD HRCT'S

Baseline volumetric HRCTs from 203 patients, acquired between 2008 and 2016, were presented to reviewers. Patient characteristics, including composite physiologic index (CPI) [15] and gender, age, and lung physiology (GAP) index [16], are detailed in Table 1. The majority of patients were men (*n* = 130, 64%), median age was 70 years (range: 32–91 years) and 141 (69%) had a history of ever smoking. Lung function was moderately impaired, with an FVC percent predicted: 79% [65–94] and a DLCO percent predicted: 47% [38–59]. One‐third of patients had been prescribed antifibrotic medications. Mean follow up time was 4.1 ± 2.5 years, during which time 139 patients died and 20 were transplanted. Radiologist assessment of HRCTs showed a total ILD extent of 31% (IQR: 21–43) and 17% and 31% were classified as ‘indeterminate for UIP’ and ‘alternative diagnosis’, respectively, based on ATS/ERS/JRS/ALAT 2018 guidelines [5].

**TABLE 1:** Patient characteristics (*N* = 203).

	Median [IQR] or *N* (%)
Age, years	70 [66–77]
Sex, M/F	130/73
FVC, % predicted	79 [65–94]
DLCO, % predicted	47 [38–59]
CPI	42 [29–53]
GAP stage 1 (%)	70 (34)
GAP stage 2 (%)	113 (56)
Gap stage 3 (%)	20 (10)
Smoking history, ever (%)	141 (69)

Abbreviations: CPI, composite physiologic index; DLCO, diffusing capacity of the lungs for carbon monoxide; FVC, forced vital capacity; GAP, gender age and lung physiology (FVC and DLCO) index.

### 3.2 Participating Reviewers

Three hundred twelve radiologists and pulmonologists from 49 countries participated in this study, *The SOFIA Project*. This diverse group included 120 radiologists, 53% with ILD expertise, and 192 pulmonologists, 27% with ILD expertise (Figure E1). Overall, reviewers had a median of 12 years of clinical experience (IQR: 7–20) and the majority were based at a university hospital (72%). Pulmonologists had more years of clinical experience compared to radiologists (14 [9–20] vs. 10 [5–16.5] years, *p* < 0.05).

### 3.3 Inter‐Observer Agreement

Inter‐observer agreement for the 4 radiological UIP patterns improved significantly after SOFIA outputs were provided (Table 2). Agreement was moderate for definite UIP (ICC_pre_ = 0.54[0.50–0.60]) and improved to good following SOFIA (ICC_post_ = 0.70[0.66–0.74]). Agreement for the remaining three categories (probable UIP, indeterminate, alternative diagnosis) was fair and improved to moderate after re‐scoring (probable UIP, ICC_pre_ = 0.30[0.27–0.35], ICC_post_ = 0.53[0.49–0.58]; indeterminate, ICC_pre_ = 0.26[0.22–0.30], ICC_post_ = 0.43[0.39–0.48]; alternative diagnosis, ICC_pre_ = 0.37[0.33–0.42], ICC_post_ = 0.53[0.48–0.58]). Agreement did not differ between radiologists and pulmonologists, between those with and without ILD expertise, or between those with the most clinical experience compared to those with the least experience (all *p* > 0.05, see Tables S1–S4).

**TABLE 2:** Inter‐observer agreement in disease pattern scores pre and post SOFIA.

		ICC	[95% CI]
Definite UIP	Pre	0.54	[0.50–0.60]
	Post	0.70*	[0.66–0.74]
Probable UIP	Pre	0.30	[0.27–0.35]
	Post	0.53*	[0.49–0.58]
Indeterminate	Pre	0.26	[0.22–0.30]
	Post	0.43*	[0.39–0.48]
Alternative Diagnosis	Pre	0.37	[0.33–0.42]
	Post	0.53*	[0.48–0.58]

*Note:* ‘*’ denotes significant pre‐ to post‐ change.

Abbreviations: ICC, intraclass correlation coefficient; UIP, usual interstitial pneumonia.

### 3.4 Impact of SOFIA on Reviewers' Scores

**FIGURE 1:**
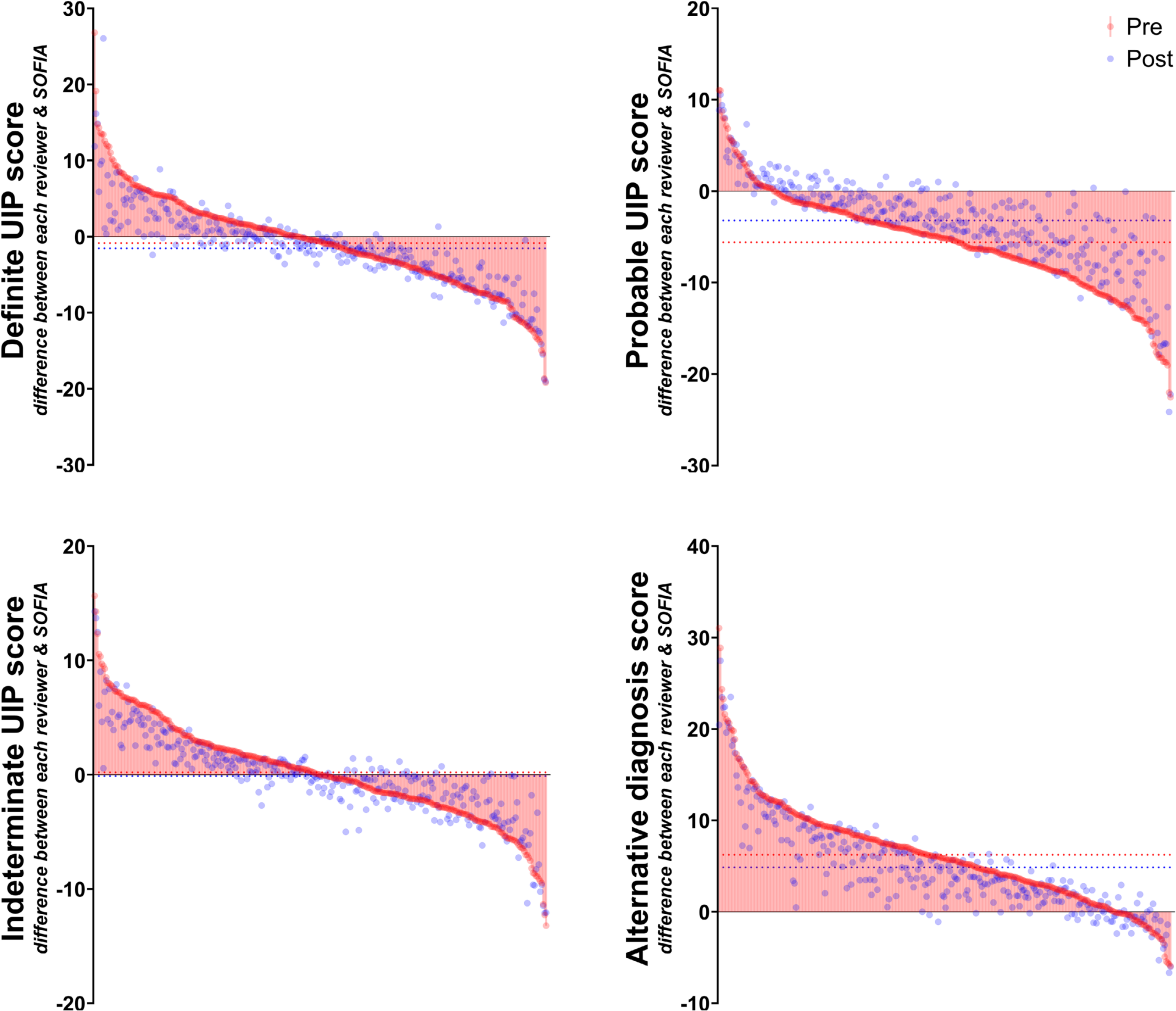
Mean difference between each reviewer's scores for all HRCT cases and SOFIA scores at baseline (red) and after viewing SOFIA scores (blue). Each blue dot that falls within the red shaded region indicates a reviewer whose revised scores were closer to SOFIA outputs than their original scores. Dotted lines represent the mean difference between all reviewers and SOFIA at baseline (red) and after viewing SOFIA scores (blue).

We explored the rationale for these improvements in agreement after viewing SOFIA outputs, and evaluated whether the improvement may be due to reviewers updating their scores to conform with SOFIA. Reviewers opted to keep their initial 4 UIP classification scores only 24% of the time. When reviewers updated their UIP scores, probable UIP was revised most frequently (80%), followed by definite UIP (67%), indeterminate (67%), and alternative diagnosis (66%). The distribution of UIP scores for each reviewer before and after viewing SOFIA outputs is presented in Figure 1. Overall, reviewers adjusted their scores to better align with SOFIA, most notably by increasing their probable UIP scores and decreasing their alternative diagnosis scores.

### 3.5 Impact of SOFIA on Prognostic Accuracy—Transplant‐Free Survival

**FIGURE 2:**
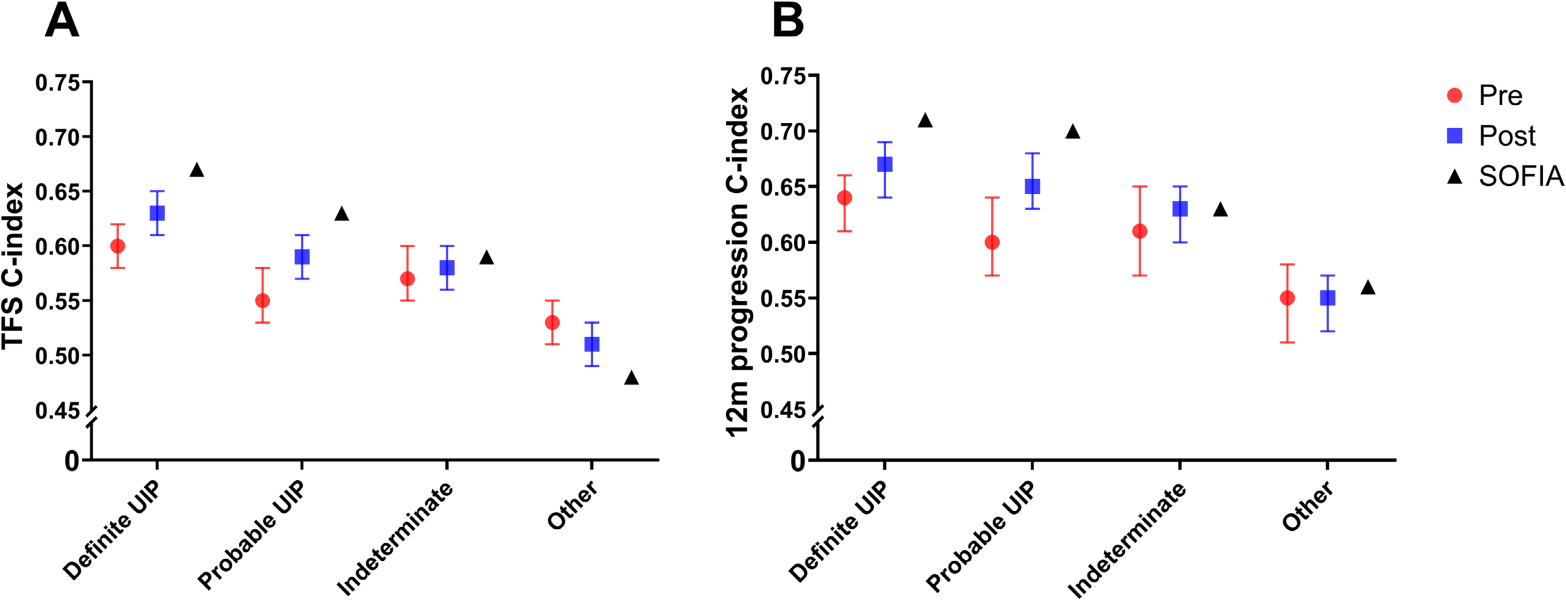
Concordance (C‐index) between reviewers' scores and (A) transplant‐free survival and (B) disease progression at 12 months before (Pre), and after (Post) viewing SOFIA scores (SOFIA). TFS, transplant‐free survival; Other, alternative diagnosis.

Before viewing SOFIA outputs, reviewers' definite UIP scores were most strongly concordant with transplant free survival (TFS, time‐to‐death or transplant, C‐index: 0.60 [0.58–0.62], Figure 2). Concordance for TFS significantly improved for definite UIP, probable UIP, and indeterminate scores, and worsened for alternative diagnosis score after viewing SOFIA outputs (Tables 3, S6, S7).

**TABLE 3:** Concordance (C‐index) between reviewers' scores and transplant‐free survival and 12‐month disease progression pre and post SOFIA.

		Transplant‐free survival		12‐month disease progression	
		Median	(IQR)	Median	(IQR)
Definite UIP	Pre	0.60	(0.58–0.62)	0.64	(0.61–0.66)
	**Post**	0.63*	**(0.61–0.65)**	0.67*	**(0.64–0.69)**
	*Change*	*0.03*	*(0.01–0.04)*	*0.03*	*(0.01–0.04)*
Probable UIP	Pre	0.55	(0.53–0.58)	0.60	(0.57–0.64)
	**Post**	0.59*	**(0.57–0.61)**	0.65*	**(0.63–0.68)**
	*Change*	*0.03*	*(0.02–0.05)*	*0.05*	*(0.02–0.07)*
Indeterminate	Pre	0.57	(0.55–0.60)	0.61	(0.57–0.65)
	**Post**	0.58*	**(0.56–0.60)**	0.63*	**(0.60–0.65)**
	*Change*	*0.01*	*(0.00–0.02)*	*0.01*	*(−0.01–0.03)*
Alternative diagnosis	Pre	0.53	(0.51–0.55)	0.55	(0.51–0.58)
	**Post**	0.51*	**(0.49–0.53)**	0.55	(0.52**–**0.57)
	*Change*	*−0.01*	*(−0.03–0.00)*	*0.00*	*(−0.03–0.03)*

*Note:* ‘*’ denotes significant pre‐ to post‐change. Bold indicates a significant change from “Pre” values. Italic indicates the change from Pre to Post.

Abbreviation: UIP, usual interstitial pneumonia.

Following SOFIA, the proportion of reviewers with probable UIP classifications which were prognostically significant increased from 28% to 70% (87 to 217 reviewers; *baseline*, median HR = 0.996[0.993–0.999], median C‐index = 0.55[0.53–0.58]; *post‐SOFIA*, median HR = 0.991[0.989–0.995], median C‐index = 0.59[0.57–0.61]). Change in TFS C‐index score was not different between reviewers with more vs. less experience (Table S8); however, there was a greater decrease in C‐index for alternative diagnosis in the group of respiratory physicians without ILD expertise (Table S5).

### 3.6 Impact of SOFIA on Prognostic Accuracy—12‐Month Disease Progression

After viewing SOFIA outputs, there was a significant improvement in the concordance of reviewers' definite UIP, probable UIP, and indeterminate scores with 12‐month disease progression, and no change in C‐index for alternative diagnosis scores (Tables 3, S9, S10; Figure 2). ILD experts had a greater improvement in concordance of probable UIP and alternative diagnosis scores and 12‐month disease progression compared to non‐experts (Table S11).

### 3.7 Application to ILD Clinical Trial Inclusion Criteria

**FIGURE 3:**
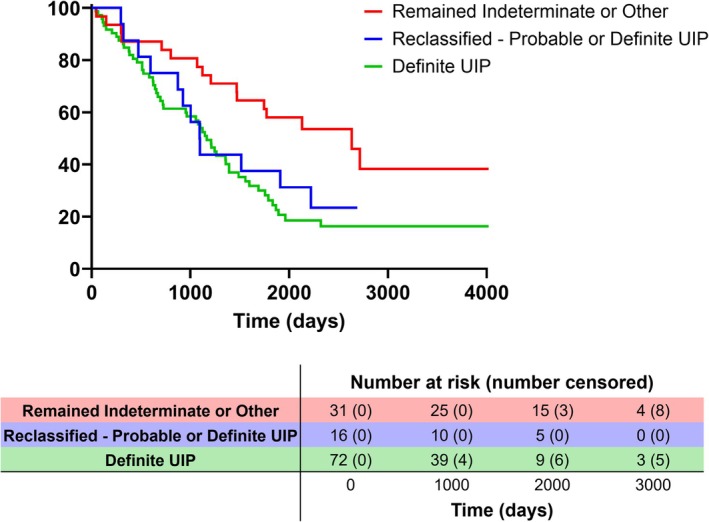
Survival differences among patients initially classified as indeterminate or alternative diagnosis by the majority of reviewers, who were then re‐classified as probable or definite UIP (blue, *N* = 16) after viewing SOFIA outputs compared to those that remained indeterminate/alternative diagnosis (red, *N* = 31; HR: 1.88 [0.86–4.13], *p* = 0.114). Survival curve for patients initially classified as definite UIP included for reference (green, *N* = 72).

We explored the influence of SOFIA in an ILD clinical trial context in which patients met inclusion criteria if they had a definite or probable UIP on HRCT. Using the baseline majority opinion of all reviewers, 47 of 203 cases (23%) were initially classified as either indeterminate (*N* = 10) or alternative diagnosis (*N* = 37), and would have been excluded from clinical trials. After implementation of SOFIA, 12 alternative diagnosis cases were re‐classified as probable (*N* = 7) or definite UIP (*N* = 5), and 4 indeterminate cases were re‐classified as probable UIP. In total, 16 of 47 patients (34%) who were initially excluded would meet trial inclusion criteria. A trend of prognostic separation between newly included patients and those who remained excluded after re‐scoring was evident (HR: 1.88 [0.86–4.13], *p* = 0.114; Figure 3).

## 4 Discussion

HRCT diagnostic classification plays a critical role in therapeutic decision‐making and clinical trial eligibility for the ILD patient [3, 4]. Despite its importance in ILD diagnosis and management, we and others have found only a fair to moderate level of agreement of HRCT UIP diagnostic classification between reviewers, suggesting that these guidelines may be applied inconsistently [2]. Following utilisation of our deep learning algorithm, SOFIA, as an automated assistive tool provided to radiologists and pulmonologists, we demonstrate improved agreement across all UIP diagnostic categories in a fibrotic ILD population. Classification accuracy, measured by concordance with clinical outcomes, was also significantly improved. To our knowledge, this is the first study of applying a deep learning algorithm for CT scans as an automated assistive diagnostic tool in a fibrotic lung disease population.

A systematic approach is recommended when applying guideline criteria to HRCTs to classify scans into one of four UIP categories [1, 17]. Quantitative or deep learning techniques can be used to automate classification of HRCTs, providing objective and reproducible outputs, and many of these tools outperform visual assessment [5, 10, 18, 19, 20, 21]. However, in practice, the implementation of such automated techniques is dependent on how clinicians view the algorithm outputs and adapt their clinical assessment accordingly. The lack of agreement between radiologists for definite UIP in our study mirrors previous findings [2, 10] and we found that inter‐observer agreement was poor for probable UIP, indeterminate, and alternative diagnosis patterns. When reviewers were given SOFIA to assist in HRCT classification, their inter‐observer agreement improved across all categories, with the greatest improvements observed for probable UIP. The impact of tools such as SOFIA depends on whether clinicians trust the information they receive from it. As SOFIA's superior performance compared to visual scoring has been validated previously, participating reviewers in this study may have been more likely to trust the algorithm. An advantage of this study was that reviewers provided granular scores for each category (0%–100% in 5% increments) rather than a single overall classification, which enabled us to detect small shifts in opinion. Our findings indicate reviewers generally trusted the algorithm and shifted their scores based on feedback from SOFIA without blindly following the algorithm. SOFIA may be useful for improving agreement between clinicians, so enabling greater consistency in diagnoses between centres.

Progressive pulmonary fibrosis describes a progressive phenotype of fibrotic ILD [22]. Patients with PPF have a similar risk of progression, trajectory of lung function decline, and treatment response as IPF patients [23, 24, 25, 26, 27, 28, 29]. Overall, we demonstrate that when utilising SOFIA, pulmonologists and radiologists' UIP scores were more strongly predictive of both TFS and 12‐month disease progression. This was most clearly demonstrated in the setting of probable UIP, with only 28% of reviewers having probable UIP scores which were prognostically significant at baseline, increasing to 70% after being shown SOFIA. Greater prognostic accuracy of probable UIP and indeterminate classifications may influence biopsy decisions and clinical trial eligibility, which directly impact patient care and outcomes.

Many recent fibrotic ILD clinical trials require central radiological review of HRCT to confirm the diagnosis. While this approach, coupled with more stringent inclusion criteria, may improve the likelihood of a positive outcome [30, 31], approximately 30%–50% of patients screened for ILD trials will not meet inclusion criteria [28, 30, 32], with screen failures frequently due to HRCTs being classified as indeterminate. In a simulated clinical trial scenario, our results suggest that of the 47 patients who would have been excluded due to alternative diagnosis or indeterminate HRCT scans, 16 (34%) would have been reclassified as probable UIP and met inclusion criteria if SOFIA had been used as an assistive tool. Incorporating SOFIA into the central review process may enable greater clinical trial recruitment without compromising trial outcomes. Additionally, an advantage of SOFIA is that the definite UIP score is a continuous variable between 0 and 1, which provides a highly sensitive marker of progression risk even in individuals whose HRCTs are considered indeterminate [5]. This may be particularly useful to enrich clinical trial enrolment, allowing selection for potentially progressive patients.

The potential benefits derived from using deep learning tools such as SOFIA may vary across centres with different levels of ILD experience and expertise [33]. Notably, in this study, we did not see major differences in accuracy or agreement between experts and non‐experts. Surprisingly, interobserver agreement for ILD expert pulmonologists was almost identical to ILD expert radiologists when using SOFIA (Table S1). This may reflect the fact that ILD clinical practice requires pulmonologists to be well‐trained in interpreting HRCTs. However, we cannot exclude the possibility of selection bias, as it is possible the clinicians who were willing to review more than 200 HRCTs were more knowledgeable or motivated to improve their HRCT interpretation skills than the general population of radiologists and pulmonologists. Moreover, during the study, participants had unlimited time to evaluate each scan, a luxury they may not be afforded in routine clinical practice, and a factor which may have minimised differences between experts and non‐experts [34]. Reproducibility and speed are key advantages of SOFIA over visual assessment by human observers, which may be particularly beneficial for clinics without experienced radiologists or with greater time constraints.

Automated evaluation of HRCTs using machine learning and other quantitative approaches has the potential to improve patient care [5, 18, 19, 35], however, implementation remains a challenge. The present study was designed to mimic a central read scenario in which radiologists are presented with HRCTs and no accompanying patient history or clinical findings and must apply current guidelines to determine whether a patient meets the radiologic inclusion criteria for a clinical trial. Our results support the use of SOFIA to improve inter‐observer agreement and accuracy among central readers. For the purpose of diagnosis, given the nature of this simulated HRCT review, it is possible clinicians were more amenable to altering their judgments based on the algorithm's feedback, and whether these trends would persist in a real clinical situation remains unclear. While previous studies focused on validating the accuracy of quantitative imaging biomarkers, future implementation efforts should explore how these tools might be ideally packaged to complement and add value to the existing clinical workflow.

Our study has several limitations. Specifically, the use of the C‐index to evaluate SOFIA's impact on reviewers' prognostic accuracy warrants further discussion. We chose to use Cox proportional hazards modelling to calculate a hazard ratio and Harrell's C‐index for each reviewer, to determine concordance of each reviewer's 203 scores with TFS. A known pitfall of the C‐index when applied to survival outcomes is its dependence on the underlying risk differences between comparable patients in the sample [36]. Achieving a high concordance with survival outcomes (a high C‐index) can be challenging when patients in the cohort have a similar risk profile of death/transplant (i.e., fibrotic ILD patients of similar ages). Indeed, in this study, a change in C‐index from 0.55 to 0.59 corresponded to a clinically meaningful shift of 130 (42%) reviewers with probable UIP scores that were concordant with transplant‐free survival *only* following access to SOFIA. Additionally, one‐third of patients were prescribed antifibrotic therapy. However, while antifibrotic use was recorded in the registry, the observational nature of the dataset precluded consistent documentation of therapy start dates, adherence, or dosage. To avoid introducing bias from incompletely captured treatment variables, we did not adjust survival or progression analyses for antifibrotic use. Future prospective studies incorporating complete treatment data will be necessary to fully assess SOFIA's impact on treatment‐mediated outcomes. In addition, this first implementation study for SOFIA involved 312 individual pulmonologists and radiologists but did not simulate a clinical setting. In practice, clinical data would be incorporated into both diagnostic and prognostic assessment, and this would likely be in the setting of an ILD specific multidisciplinary meeting (MDM). Indeed, incorporating SOFIA as an automated assistive tool into the ILD MDM, as well as clinical trial central‐read settings are the next logical step towards implementation.

In conclusion, this study represents an initial step towards the incorporation of a validated deep learning automated assistive tool into ILD clinical care. We show improvements in inter‐observer agreement for UIP classification, as well as concordance with clinical outcomes following SOFIA. Future prospective research and implementation efforts are needed to determine SOFIA's influence on multidisciplinary team decisions, the gold standard for ILD diagnosis and management.

## Author Contributions

**Caitlin C. Fermoyle:** writing – original draft, visualization, methodology, writing – review and editing, formal analysis, validation, funding acquisition. **John A. Mackintosh:** visualization, writing – review and editing, data curation, project administration. **Vidya Navaratnam:** writing – review and editing, data curation, project administration. **Samantha Ellis:** writing – review and editing, data curation, project administration. **Wendy A. Cooper:** writing – review and editing, data curation, project administration. **Nicole Goh:** writing – review and editing, data curation, project administration. **Yuben Moodley:** writing – review and editing, data curation, project administration. **Paul N. Reynolds:** writing – review and editing, data curation, project administration. **Christopher J. Zappala:** writing – review and editing, data curation, project administration. **Peter Hopkins:** writing – review and editing, data curation, project administration. **Ian N. Glaspole:** writing – review and editing, data curation, project administration. **Tamera J. Corte:** resources, supervision, writing – review and editing, visualization, methodology, funding acquisition, project administration, validation. **Simon L. F. Walsh:** conceptualization, investigation, funding acquisition, methodology, validation, visualization, writing – review and editing, software, formal analysis, project administration, resources, supervision, data curation.

## Funding

The Australian IPF Registry is an initiative of Lung Foundation Australia and is supported by Foundation partners Boehringer Ingelheim, Roche Products Pty. Limited. C.C.F. is supported by a fellowship from Lung Foundation Australia & the Centre of Research Excellence in Pulmonary Fibrosis (CRE‐PF; CREATE HOPE Fellowship) and the National Health and Medical Research Council (NHMRC Investigator Grant 2017479 awarded to T.J.C.).

## Ethics Statement

The use of anonymised clinical data and images for this study was approved by Sydney Local Health District ethics committee (protocol X14‐0264).

## Conflicts of Interest

Unrelated to the current work, T.J.C. reports grants or contracts from Boehringer Ingleheim, Pharmaxis, Bristol Myers Squibb, 4D, Roche, Pliant, Bridge Biotherapeutics and Avalyn Therapeutics; consulting fees from Boehringer Ingleheim, Pharmaxis, Bristol Myers Squibb, Ad Alta, Roche, Pliant, Bridge Biotherapeutics, Avalyn Therapeutics, DevPro, Endeavour BioMedicine; honoraria for lectures, presentations, speakers bureaus, manuscript writing or educational events from Bristol Myers Squibb, Roche and Boehringer Ingleheim; support for attending meetings and/or travel with Bristol Myers Squibb and Boehringer Ingelheim; participation on a data safety monitoring board or advisory board with Boehringer Ingleheim, Bristol Myers Squibb, Roche, Ad Alta, Pliant, Bridge Biotherapeutics, Avalyn Therapeutics, DevPro and Endeavour BioMedicine. C.C.F. reports a grant from Lung Foundation Australia. S.L.F.W. reports grants or contracts from Roche, Pliant, Avalyn Therapeutics, Boehringer Ingelheim, and Qureight Ltd.; consulting fees from Pliant, Avalyn Therapeutics, DevPro, Endeavour BioMedicine; honoraria for lectures, presentations, speakers bureaus, manuscript writing or educational events from Boehringer Ingelheim; stock or stock options from Qureight Ltd. V.N. reports consulting fees from Boehringer Ingelheim; honoraria for lectures, presentations, speakers bureaus, manuscript writing or educational events from Boehringer Ingelheim; support for attending meetings and/or travel from Boehringer Ingelheim and Bristol Myers Squibs. J.A.M. reports honoraria for lectures, presentations, speakers bureaus, manuscript writing or educational events and support for attending meetings and/or travel from Boehringer Ingelheim. I.N.G. reports participation on a data safety monitoring board or advisory board with TianLi Pharmaceuticals, Pliant, Avalyn Therapeutics, Endeavour BioMedicines, Accendatech; holds a leadership or fiduciary role in the Pulmonary Fibrosis Australasian Clinical Trials Network and Australian ILD registry. P.H. reports leadership or fiduciary role in the International Society of Heart and Lung Transplantation. N.S.L.G. reports consulting fees from Boehringer Ingelheim; honoraria for lectures, presentations, speakers bureaus from Boehringer Ingelheim and AstraZeneca; support for attending meetings and/or travel from Boehringer Ingelheim and Chiesi; participation on a data safety monitoring board or advisory board from Boehringer Ingelheim; holds leadership or fiduciary roles in the Thoracic Society of Australia and New Zealand; and reports the receipt of equipment and materials from Air Liquide (portable oxygen concentrators). N.G., T.J.C. and Y.M. are Editorial Board members of Respirology and co‐authors of this article. They were excluded from all editorial decision‐making related to the acceptance of this article for publication.

## Supporting information


**Table S1:** Inter‐observer agreement for the 4 radiological patterns before (pre) and after (post) viewing SOFIA outputs for all reviewers, general pulmonologists without ILD expertise, general radiologists without ILD expertise, pulmonologists with ILD expertise, and radiologists with ILD expertise.
**Table S2:** Inter‐observer agreement for the 4 radiological patterns before (pre) and after (post) viewing SOFIA outputs for radiologists and pulmonologists.
**Table S3:** Inter‐observer agreement for the 4 radiological patterns before (pre) and after (post) viewing SOFIA outputs for reviewers with and without ILD expertise.
**Table S4:** Inter‐observer agreement for the 4 radiological patterns before (pre) and after (post) viewing SOFIA outputs for reviewers with the most experience (top quartile) and least experience (bottom quartile).
**Table S5:** Change in concordance (c‐index) between each reviewer's scores and transplant‐survival before (pre) and after (post) viewing SOFIA outputs by group—all reviewers, general pulmonologists, general radiologists, pulmonologists with ILD expertise, and radiologists with ILD expertise.
**Table S6:** Change in concordance (c‐index) between each reviewer's scores and transplant‐survival before (pre) and after (post) viewing SOFIA outputs for radiologists and pulmonologists.
**Table S7:** Change in concordance (c‐index) between each reviewer's scores and transplant‐survival before (pre) and after (post) viewing SOFIA outputs for reviewers with and without ILD expertise.
**Table S8:** Change in concordance (c‐index) between each reviewer's scores and transplant‐survival before (pre) and after (post) viewing SOFIA outputs for reviewers with the greatest and least number of years of experience (top and bottom quartile, respectively).
**Table S9:** Change in concordance (c‐index) between each reviewer's scores and 12‐month disease progression before (pre) and after (post) viewing SOFIA outputs by group—all reviewers, general pulmonologists, general radiologists, pulmonologists with ILD expertise, and radiologists with ILD expertise.
**Table S10:** Change in concordance (c‐index) between each reviewer's scores and 12‐month disease progression before (pre) and after (post) viewing SOFIA outputs for radiologists and pulmonologists.
**Table S11:** Change in concordance (c‐index) between each reviewer's scores and 12‐month disease progression before (pre) and after (post) viewing SOFIA outputs for reviewers with and without ILD expertise.
**Table S12:** Change in concordance (c‐index) between each reviewer's scores and 12‐month disease progression before (pre) and after (post) viewing SOFIA outputs for reviewers with the greatest and least number of years of experience (top and bottom quartile, respectively).
**Figure S1:** Distribution of reviewer locations by country.

**Data S1:** The SOFIA Project Consortium.

## Data Availability

The data that support the findings of this study are available on request from the corresponding author. The data are not publicly available due to privacy or ethical restrictions.

## References

[1] G. Raghu , M. Remy‐Jardin , J. L. Myers , et al., “Diagnosis of Idiopathic Pulmonary Fibrosis an Official ATS/ERS/JRS/ALAT Clinical Practice Guideline,” American Journal of Respiratory and Critical Care Medicine 198 (2018): e44–e68. 30168753 10.1164/rccm.201807-1255ST

[2] S. L. F. Walsh , L. Calandriello , N. Sverzellati , A. U. Wells , D. M. Hansell , and UIP Observer Consort , “Interobserver Agreement for the ATS/ERS/JRS/ALAT Criteria for a UIP Pattern on CT,” Thorax 71 (2016): 45–51. 26585524 10.1136/thoraxjnl-2015-207252

[3] K. R. Flaherty , A. C. Andrei , T. E. King , et al., “Idiopathic Interstitial Pneumonia: Do Community and Academic Physicians Agree on Diagnosis?,” American Journal of Respiratory and Critical Care Medicine 175 (2007): 1054–1060. 17255566 10.1164/rccm.200606-833OC PMC1899268

[4] L. Richeldi , R. M. du Bois , G. Raghu , et al., “Efficacy and Safety of Nintedanib in Idiopathic Pulmonary Fibrosis,” New England Journal of Medicine 370 (2014): 2071–2082. 24836310 10.1056/NEJMoa1402584

[5] S. L. F. Walsh , J. A. Mackintosh , L. Calandriello , et al., “Deep Learning‐Based Outcome Prediction in Progressive Fibrotic Lung Disease Using High‐Resolution Computed Tomography,” American Journal of Respiratory and Critical Care Medicine 206 (2022): 883–891. 35696341 10.1164/rccm.202112-2684OC

[6] H. E. Jo , I. Glaspole , Y. Moodley , et al., “Disease Progression in Idiopathic Pulmonary Fibrosis With Mild Physiological Impairment: Analysis From the Australian IPF Registry,” BMC Pulmonary Medicine 18 (2018): 19. 29370786 10.1186/s12890-018-0575-y PMC5785886

[7] H. E. Jo , I. Glaspole , C. Grainge , et al., “Baseline Characteristics of Idiopathic Pulmonary Fibrosis: Analysis From the Australian Idiopathic Pulmonary Fibrosis Registry,” European Respiratory Journal 49 (2017): 1601592. 28232409 10.1183/13993003.01592-2016

[8] G. Raghu , H. R. Collard , J. J. Egan , et al., “An Official ATS/ERS/JRS/ALAT Statement: Idiopathic Pulmonary Fibrosis: Evidence‐Based Guidelines for Diagnosis and Management,” American Journal of Respiratory and Critical Care Medicine 183 (2011): 788–824. 21471066 10.1164/rccm.2009-040GL PMC5450933

[9] C. Szegedy , S. Ioffe , V. Vanhoucke , and A. Alemi , “Inception‐v4, Inception‐ResNet and the Impact of Residual Connections on Learning,” Proceedings of the AAAI Conference on Artificial Intelligence 31 (2017): 4278–4284.

[10] S. L. F. Walsh , L. Calandriello , M. Silva , and N. Sverzellati , “Deep Learning for Classifying Fibrotic Lung Disease on High‐Resolution Computed Tomography: A Case‐Cohort Study,” Lancet Respiratory Medicine 6 (2018): 837–845. 30232049 10.1016/S2213-2600(18)30286-8

[11] J. L. Fleiss and J. Cohen , “The Equivalence of Weighted Kappa and the Intraclass Correlation Coefficient as Measures of Reliability,” Educational and Psychological Measurement 33 (1973): 613–619.

[12] P. Brennan and A. Silman , “Statistical Methods for Assessing Observer Variability in Clinical Measures,” BMJ 304 (1992): 1491–1494. 1611375 10.1136/bmj.304.6840.1491 PMC1882212

[13] F. E. Harrell , K. L. Lee , and D. B. Mark , “Multivariable Prognostic Models: Issues in Developing Models, Evaluating Assumptions and Adequacy, and Measuring and Reducing Errors,” Statistics in Medicine 15 (1996): 361–387. 8668867 10.1002/(SICI)1097-0258(19960229)15:4<361::AID-SIM168>3.0.CO;2-4

[14] J. V. Pugashetti , A. Adegunsoye , Z. Wu , et al., “Validation of Proposed Criteria for Progressive Pulmonary Fibrosis,” American Journal of Respiratory and Critical Care Medicine 207 (2023): 69–76. 35943866 10.1164/rccm.202201-0124OC PMC9952866

[15] A. U. Wells , S. R. Desai , M. B. Rubens , et al., “Idiopathic Pulmonary Fibrosis: A Composite Physiologic Index Derived From Disease Extent Observed by Computed Tomography,” American Journal of Respiratory and Critical Care Medicine 167 (2003): 962–969. 12663338 10.1164/rccm.2111053

[16] B. Ley , C. J. Ryerson , E. Vittinghoff , et al., “A Multidimensional Index and Staging System for Idiopathic Pulmonary Fibrosis,” Annals of Internal Medicine 156 (2012): 684–691. 22586007 10.7326/0003-4819-156-10-201205150-00004

[17] D. A. Lynch , N. Sverzellati , W. D. Travis , et al., “Diagnostic Criteria for Idiopathic Pulmonary Fibrosis: A Fleischner Society White Paper,” Lancet Respiratory Medicine 6 (2018): 138–153. 29154106 10.1016/S2213-2600(17)30433-2

[18] S. M. Humphries , J. A. Mackintosh , H. E. Jo , et al., “Quantitative Computed Tomography Predicts Outcomes in Idiopathic Pulmonary Fibrosis,” Respirology 27 (2022): 1045–1053. 35875881 10.1111/resp.14333 PMC9796832

[19] J. Jacob , B. J. Bartholmai , S. Rajagopalan , et al., “Automated Quantitative Computed Tomography Versus Visual Computed Tomography Scoring in Idiopathic Pulmonary Fibrosis: Validation Against Pulmonary Function,” Journal of Thoracic Imaging 31 (2016): 304–311. 27262146 10.1097/RTI.0000000000000220

[20] J. Jacob , B. J. Bartholmai , S. Rajagopalan , et al., “Mortality Prediction in Idiopathic Pulmonary Fibrosis: Evaluation of Computer‐Based CT Analysis With Conventional Severity Measures,” European Respiratory Journal 49 (2017): 1601011. 27811068 10.1183/13993003.01011-2016

[21] A. S. Oh , D. A. Lynch , J. J. Swigris , et al., “Deep Learning‐Based Fibrosis Extent on Computed Tomography Predicts Outcome of Fibrosing Interstitial Lung Disease Independent of Visually Assessed Computed Tomography Pattern,” Annals of the American Thoracic Society 21 (2024): 218–227. 37696027 10.1513/AnnalsATS.202301-084OC

[22] S. K. Rajan , V. Cottin , R. Dhar , et al., “Progressive Pulmonary Fibrosis: An Expert Group Consensus Statement,” European Respiratory Journal 61 (2023): 2103187. 36517177 10.1183/13993003.03187-2021 PMC10060665

[23] J. Jacob , N. Hirani , C. H. M. van Moorsel , et al., “Predicting Outcomes in Rheumatoid Arthritis Related Interstitial Lung Disease,” European Respiratory Journal 53 (2019): 1800869. 30487199 10.1183/13993003.00869-2018 PMC6319797

[24] C. Chan , C. J. Ryerson , J. V. Dunne , and P. G. Wilcox , “Demographic and Clinical Predictors of Progression and Mortality in Connective Tissue Disease‐Associated Interstitial Lung Disease: A Retrospective Cohort Study,” BMC Pulmonary Medicine 19 (2019): 192. 31672127 10.1186/s12890-019-0943-2 PMC6824100

[25] H. P. Fainberg , J. M. Oldham , P. L. Molyneau , et al., “Forced Vital Capacity Trajectories in Patients With Idiopathic Pulmonary Fibrosis: A Secondary Analysis of a Multicentre, Prospective, Observational Cohort,” Lancet Digital Health 4 (2022): e862–e872. 36333179 10.1016/S2589-7500(22)00173-X PMC12646456

[26] M. J. Strand , D. Sprunger , G. P. Cosgrove , et al., “Pulmonary Function and Survival in Idiopathic vs Secondary Usual Interstitial Pneumonia,” Chest 146 (2014): 775–785. 24700149 10.1378/chest.13-2388 PMC4151362

[27] A. Adegunsoye , J. M. Oldham , S. K. Bellam , et al., “Computed Tomography Honeycombing Identifies a Progressive Fibrotic Phenotype With Increased Mortality Across Diverse Interstitial Lung Diseases,” Annals of the American Thoracic Society 16 (2019): 580–588. 30653927 10.1513/AnnalsATS.201807-443OC PMC6491052

[28] K. R. Flaherty , A. U. Wells , V. Cottin , et al., “Nintedanib in Progressive Fibrosing Interstitial Lung Diseases,” New England Journal of Medicine 381 (2019): 1718–1727. 31566307 10.1056/NEJMoa1908681

[29] J. Choe , E. J. Chae , Y. J. Kim , K.‐H. Do , J. S. Song , and J. W. Song , “Serial Changes of CT Findings in Patients With Chronic Hypersensitivity Pneumonitis: Imaging Trajectories and Predictors of Fibrotic Progression and Acute Exacerbation,” European Radiology 31 (2021): 3993–4003. 33241510 10.1007/s00330-020-07469-2

[30] T. E. King , W. Z. Bradford , S. Castro‐Bernardini , et al., “A Phase 3 Trial of Pirfenidone in Patients With Idiopathic Pulmonary Fibrosis,” New England Journal of Medicine 370 (2014): 2083–2092. 24836312 10.1056/NEJMoa1402582

[31] K. K. Brown and A. U. Wells , “Recent Clinical Trials in Idiopathic Pulmonary Fibrosis and the BUILD‐1 Study,” European Respiratory Review 17 (2008): 116–122.

[32] Y. H. Khor , K. A. Johannson , V. Marcoux , et al., “Generalisability of Pharmaceutical Randomised Controlled Trial Eligibility Criteria for Progressive Pulmonary Fibrosis,” European Respiratory Journal 65 (2025): 2401575. 39510557 10.1183/13993003.01575-2024

[33] A. U. Wells , “The Revised ATS/ERS/JRS/ALAT Diagnostic Criteria for Idiopathic Pulmonary Fibrosis (IPF)—Practical Implications,” Respiratory Research 14, no. Suppl 1 (2013): S2. 23734820 10.1186/1465-9921-14-S1-S2 PMC3643186

[34] L. R. Salkowski and R. Russ , “Cognitive Processing Differences of Experts and Novices When Correlating Anatomy and Cross‐Sectional Imaging,” Journal of Medical Imaging 5 (2018): 031411. 29795777 10.1117/1.JMI.5.3.031411 PMC5958290

[35] S. M. Humphries , K. Yagihashi , J. Huckleberry , et al., “Idiopathic Pulmonary Fibrosis: Data‐Driven Textural Analysis of Extent of Fibrosis at Baseline and 15‐Month Follow‐Up,” Radiology 285 (2017): 270–278. 28493789 10.1148/radiol.2017161177 PMC5621716

[36] N. Hartman , S. Kim , K. He , and J. D. Kalbfleisch , “Pitfalls of the Concordance Index for Survival Outcomes,” Statistics in Medicine 42 (2023): 2179–2190. 36977424 10.1002/sim.9717 PMC10219847

