# Supplementary material for: Deep‐Learning Algorithm Diagnostic Support for Usual Interstitial Pneumonia Pattern Recognition in Fibrotic Interstitial Lung Disease
Source: Respirology. 2026 Apr 1;31(7):711–20. doi: 10.1002/resp.70246 (PMC13342441; doi:10.1002/resp.70246)
Supplement: Supplementary file 1 — Table S1: Inter‐observer agreement for the 4 radiological patterns before (pre) and after (post) viewing SOFIA outputs for all reviewers, general pulmonologists without ILD expertise, general radiologists without ILD expertise, pulmonologists with ILD expertise, and radiologists with ILD expertise. Table S2: Inter‐observer agreement for the 4 radiological patterns before (pre) and after (post) viewing SOFIA outputs for radiologists and pulmonologists. Table S3: Inter‐observer agreement for the 4 radiological patterns before (pre) and after (post) viewing SOFIA outputs for reviewers with and without ILD expertise. Table S4: Inter‐observer agreement for the 4 radiological patterns before (pre) and after (post) viewing SOFIA outputs for reviewers with the most experience (top quartile) and least experience (bottom quartile). Table S5: Change in concordance (c‐index) between each reviewer's scores and transplant‐survival before (pre) and after (post) viewing SOFIA outputs by group—all reviewers, general pulmonologists, general radiologists, pulmonologists with ILD expertise, and radiologists with ILD expertise. Table S6: Change in concordance (c‐index) between each reviewer's scores and transplant‐survival before (pre) and after (post) viewing SOFIA outputs for radiologists and pulmonologists. Table S7: Change in concordance (c‐index) between each reviewer's scores and transplant‐survival before (pre) and after (post) viewing SOFIA outputs for reviewers with and without ILD expertise. Table S8: Change in concordance (c‐index) between each reviewer's scores and transplant‐survival before (pre) and after (post) viewing SOFIA outputs for reviewers with the greatest and least number of years of experience (top and bottom quartile, respectively). Table S9: Change in concordance (c‐index) between each reviewer's scores and 12‐month disease progression before (pre) and after (post) viewing SOFIA outputs by group—all reviewers, general pulmonologists, general radiologists, pulm [file RESP-31-711-s002.pdf]

Online Data Supplement

Table S1. Inter-observer agreement for the 4 radiological patterns before (pre) and after (post) viewing SOFIA outputs for all reviewers, general pulmonologists without ILD expertise, general radiologists without ILD expertise, pulmonologists with ILD expertise, and radiologists with ILD expertise.

		**ALL**			**General Pulmonologists**			**General Radiologists**			**ILD Pulmonologists**			**ILD Radiologists**		
		ICC	[95% CI]		ICC	[95% CI]		ICC	[95% CI]		ICC	[95% CI]		ICC	[95% CI]	
**Definite UIP**	pre	0.54	0.50	0.60	0.52	0.47	0.57	0.53	0.48	0.58	0.56	0.51	0.61	0.61	0.56	0.66
	post	**0.70***	0.66	0.74	**0.71***	0.67	0.75	**0.68***	0.64	0.73	**0.70***	0.66	0.75	**0.72***	0.68	0.76
**Probable UIP**	pre	0.30	0.27	0.35	0.27	0.23	0.31	0.30	0.26	0.34	0.32	0.28	0.37	0.36	0.32	0.41
	post	**0.53***	0.49	0.58	**0.56***	0.51	0.61	**0.51***	0.46	0.56	**0.53***	0.48	0.58	**0.54***	0.49	0.59
**Indeterminate**	pre	0.26	0.22	0.30	0.27	0.23	0.31	0.21	0.18	0.25	0.27	0.23	0.31	0.29	0.25	0.33
	post	**0.43***	0.39	0.48	**0.48***	0.43	0.53	**0.38***	0.34	0.44	**0.43***	0.38	0.48	**0.42***	0.37	0.47
**Alternative Diagnosis**	pre	0.37	0.33	0.42	0.34	0.30	0.39	0.34	0.29	0.39	0.41	0.36	0.46	0.44	0.39	0.49
	post	**0.53***	0.48	0.58	**0.54***	0.49	0.59	**0.51***	0.46	0.56	**0.54***	0.49	0.59	**0.55***	0.51	0.61

Agreement was compared using intraclass correlation coefficients (ICC) with two-way random effects and absolute agreement. An ICC of 0-0.2 was considered poor, 0.21-0.4 fair, 0.41-0.6 moderate, 0.61-0.8 good, and >0.8 very good. ICCs with non-overlapping confidence intervals were considered to be significantly different. *Indicates pre to post difference in ICCs.

Table S2. Inter-observer agreement for the 4 radiological patterns before (pre) and after (post) viewing SOFIA outputs for radiologists and pulmonologists.

		Radiologists			Pulmonologists		
		ICC	[95% CI]		ICC	[95% CI]	
**Definite UIP**	pre	0.57	0.52	0.62	0.53	0.49	0.58
	post	**0.70***	0.66	0.74	**0.71***	0.66	0.75
**Probable UIP**	pre	0.32	0.28	0.37	0.29	0.25	0.34
	post	**0.52***	0.47	0.57	**0.54***	0.50	0.59
**Indeterminate**	pre	0.24	0.21	0.29	0.27	0.23	0.31
	post	**0.40***	0.35	0.45	**0.46***	0.41	0.51
**Alternative Diagnosis**	pre	0.38	0.34	0.43	0.37	0.33	0.42
	post	**0.52***	0.48	0.58	**0.53***	0.49	0.59

Agreement was compared using intraclass correlation coefficients (ICC) with two-way random effects and absolute agreement. An ICC of 0-0.2 was considered poor, 0.21-0.4 fair, 0.41-0.6 moderate, 0.61-0.8 good, and >0.8 very good. ICCs with non-overlapping confidence intervals were considered to be significantly different. *Indicates pre to post difference in ICCs.

Table S3. Inter-observer agreement for the 4 radiological patterns before (pre) and after (post) viewing SOFIA outputs for reviewers with and without ILD expertise.

		Reviewers with ILD expertise			Reviewers without ILD expertise		
		ICC	[95% CI]		ICC	[95% CI]	
**Definite UIP**	pre	0.58	0.53	0.63	0.52	0.48	0.57
	post	**0.71***	0.67	0.75	**0.70***	0.66	0.74
**Probable UIP**	pre	0.34	0.30	0.39	0.28	0.24	0.32
	post	**0.53***	0.49	0.58	**0.54***	0.49	0.59
**Indeterminate**	pre	0.27	0.24	0.32	0.24	0.21	0.29
	post	**0.43***	0.38	0.48	**0.44***	0.39	0.49
**Alternative Diagnosis**	pre	0.42	0.37	0.47	0.34	0.30	0.39
	post	**0.54***	0.50	0.59	**0.52***	0.48	0.57

Agreement was compared using intraclass correlation coefficients (ICC) with two-way random effects and absolute agreement. An ICC of 0-0.2 was considered poor, 0.21-0.4 fair, 0.41-0.6 moderate, 0.61-0.8 good, and >0.8 very good. ICCs with non-overlapping confidence intervals were considered to be significantly different. *Indicates pre to post difference in ICCs.

Table S4. Inter-observer agreement for the 4 radiological patterns before (pre) and after (post) viewing SOFIA outputs for reviewers with the most experience (top quartile) and least experience (bottom quartile).

		**Most experienced reviewers**			**Least experienced reviewers**		
		ICC	[95% CI]		ICC	[95% CI]	
**Definite UIP**	pre	0.54	0.49	0.59	0.54	0.50	0.60
	post	**0.69***	0.64	0.73	**0.72***	0.68	0.76
**Probable UIP**	pre	0.29	0.25	0.34	0.32	0.28	0.37
	post	**0.51***	0.46	0.56	**0.56***	0.52	0.61
**Indeterminate**	pre	0.24	0.21	0.28	0.28	0.25	0.33
	post	**0.41***	0.37	0.47	**0.48***	0.43	0.53
**Alternative Diagnosis**	pre	0.39	0.34	0.44	0.36	0.32	0.41
	post	**0.54***	0.50	0.60	**0.54***	0.50	0.60

Agreement was compared using intraclass correlation coefficients (ICC) with two-way random effects and absolute agreement. An ICC of 0-0.2 was considered poor, 0.21-0.4 fair, 0.41-0.6 moderate, 0.61-0.8 good, and >0.8 very good. ICCs with non-overlapping confidence intervals were considered to be significantly different. *Indicates pre to post difference in ICCs.

Table S5. Change in concordance (c-index) between each reviewer's scores and transplant-survival before (pre) and after (post) viewing SOFIA outputs by group – all reviewers, general pulmonologists, general radiologists, pulmonologists with ILD expertise, and radiologists with ILD expertise.

		**ALL**			**General Pulmonologists**			**General Radiologists**			**ILD Pulmonologists**			**ILD Radiologists**		
		Median	IQR		Median	IQR		Median	IQR		Median	IQR		Median	IQR	
**Definite UIP**	pre	0.60	0.58	0.62	0.61	0.59	0.62	0.60	0.58	0.62	0.60	0.58	0.62	0.60	0.58	0.61
	**post**	**0.63***	**0.61**	**0.65**	**0.64***	**0.62**	**0.66**	**0.63***	**0.61**	**0.65**	**0.63***	**0.61**	**0.65**	**0.62***	**0.61**	**0.64**
	change	0.03	0.01	0.04	0.03	0.01	0.04	0.02	0.01	0.04	0.03	0.02	0.04	0.03	0.01	0.03
**Probable UIP**	pre	0.55	0.53	0.58	0.55	0.52	0.58	0.55	0.53	0.58	0.55	0.52	0.57	0.55	0.53	0.57
	**post**	**0.59***	**0.57**	**0.61**	**0.60***	**0.57**	**0.61**	**0.59***	**0.57**	**0.61**	**0.59***	**0.57**	**0.61**	**0.59***	**0.57**	**0.60**
	change	0.03	0.02	0.05	0.04	0.02	0.06	0.03	0.01	0.05	0.03	0.02	0.05	0.03	0.02	0.04
**Indeterminate**	pre	0.57	0.55	0.60	0.59	0.55	0.61	0.56	0.54	0.59	0.57	0.54	0.60	0.57	0.55	0.59
	**post**	**0.58***	**0.56**	**0.60**	**0.59***	**0.58**	**0.61**	**0.57***	**0.55**	**0.59**	**0.58***	**0.56**	**0.60**	**0.58***	**0.56**	**0.59**
	change	0.01	0.00	0.02	0.00	0.00	0.02	0.01	-0.01	0.02	0.01	0.00	0.02	0.01	0.00	0.02
**Alternative diagnosis**	pre	0.53	0.51	0.55	0.53	0.51	0.55	0.53	0.51	0.56	0.52	0.50	0.54	0.52	0.51	0.55
	**post**	**0.51***	**0.49**	**0.53**	**0.51***	**0.49**	**0.53**	**0.52***	**0.50**	**0.54**	**0.51***	**0.49**	**0.53**	**0.51***	**0.49**	**0.52**
	change	-0.01	-0.03	0.00	-0.02†	-0.04	0.00	-0.01	-0.03	0.00	-0.01	-0.03	0.00	-0.01	-0.03	-0.01

Pre- to post- change in C-index was compared using a Wilcoxon signed rank test, and between group differences in change scores were compared using a Kruskal-Wallis test (4 groups) with a significance level of $p<0.05$. *Indicates a significant difference pre to post; †Indicates significant group differences in change scores.

Table S6. Change in concordance (c-index) between each reviewer's scores and transplant-survival before (pre) and after (post) viewing SOFIA outputs for radiologists and pulmonologists.

		**Radiologists**			**Pulmonologists**		
		Median	IQR		Median	IQR	
**Definite UIP**	pre	0.60	0.58	0.61	0.61	0.58	0.62
	**post**	**0.62***	**0.61**	**0.64**	**0.64***	**0.62**	**0.65**
	change	0.02	0.01	0.04	0.03	0.02	0.04
**Probable UIP**	pre	0.55	0.53	0.57	0.55	0.52	0.58
	**post**	**0.59***	**0.57**	**0.61**	**0.60***	**0.57**	**0.61**
	change	0.03	0.01	0.05	0.03	0.02	0.06
**Indeterminate**	pre	0.57	0.54	0.59	0.58	0.55	0.60
	**post**	**0.58***	**0.56**	**0.59**	**0.59***	**0.57**	**0.61**
	change	0.01	0.00	0.02	0.01	0.00	0.02
**Alternative diagnosis**	pre	0.52	0.51	0.55	0.53	0.51	0.55
	**post**	**0.51***	**0.49**	**0.53**	**0.51***	**0.49**	**0.53**
	change	-0.01	-0.03	0.00	-0.01	-0.04	0.00

Pre- to post- change in C-index was compared using a Wilcoxon signed rank test, and between group differences in change scores were compared using a Mann-Whitney U test (2 groups) with a significance level of $p<0.05$. *Indicates a significant difference pre to post; †Indicates significant group differences in change scores.

Table S7. Change in concordance (c-index) between each reviewer's scores and transplant-survival before (pre) and after (post) viewing SOFIA outputs for reviewers with and without ILD expertise.

		**Reviewers with ILD expertise**			**Reviewers without ILD expertise**		
		Median	IQR		Median	IQR	
**Definite UIP**	pre	0.60	0.58	0.62	0.60	0.59	0.62
	**post**	**0.63***	**0.61**	**0.64**	**0.64***	**0.62**	**0.65**
	change	0.03	0.02	0.03	0.02	0.01	0.04
**Probable UIP**	pre	0.55	0.52	0.57	0.55	0.53	0.58
	**post**	**0.59***	**0.57**	**0.61**	**0.60***	**0.57**	**0.61**
	change	0.03	0.02	0.05	0.04	0.01	0.06
**Indeterminate**	pre	0.57	0.54	0.59	0.58	0.55	0.60
	**post**	**0.58***	**0.56**	**0.60**	**0.59***	**0.56**	**0.61**
	change	0.01	0.00	0.02	0.00	0.00	0.02
**Alternative diagnosis**	pre	0.52	0.50	0.54	0.53	0.51	0.55
	**post**	**0.51***	**0.49**	**0.53**	**0.51***	**0.49**	**0.54**
	change	-0.01	-0.03	0.00	-0.02	-0.04	0.00

Pre- to post- change in C-index was compared using a Wilcoxon signed rank test, and between group differences in change scores were compared using a Mann-Whitney U test (2 groups) with a significance level of $p<0.05$. *Indicates a significant difference pre to post; †Indicates significant group differences in change scores.

Table S8. Change in concordance (c-index) between each reviewer's scores and transplant-survival before (pre) and after (post) viewing SOFIA outputs for reviewers with the greatest and least number of years of experience (top and bottom quartile, respectively).

		**Most experienced reviewers**			**Least experienced reviewers**		
		Median	IQR		Median	IQR	
**Definite UIP**	pre	0.60	0.58	0.62	0.60	0.59	0.62
	**post**	**0.63***	**0.61**	**0.65**	**0.64***	**0.62**	**0.65**
	change	0.02	0.01	0.04	0.02	0.01	0.03
**Probable UIP**	pre	0.55	0.52	0.58	0.55	0.54	0.57
	**post**	**0.59***	**0.57**	**0.61**	**0.59***	**0.57**	**0.61**
	change	0.03	0.02	0.05	0.03	0.02	0.05
**Indeterminate**	pre	0.57	0.54	0.60	0.58	0.55	0.60
	**post**	**0.58***	**0.55**	**0.60**	**0.59***	**0.57**	**0.61**
	change	0.00	0.00	0.01	0.01	0.00	0.02
**Alternative diagnosis**	pre	0.53	0.51	0.55	0.53	0.51	0.55
	**post**	**0.52***	**0.49**	**0.54**	**0.51***	**0.49**	**0.53**
	change	-0.01	-0.03	0.00	-0.02	-0.03	0.00

Pre- to post- change in C-index was compared using a Wilcoxon signed rank test, and between group differences in change scores were compared using a Mann-Whitney U test (2 groups) with a significance level of $p<0.05$. *Indicates a significant difference pre to post; †Indicates significant group differences in change scores.

Table S9. Change in concordance (c-index) between each reviewer's scores and 12-month disease progression before (pre) and after (post) viewing SOFIA outputs by group – all reviewers, general pulmonologists, general radiologists, pulmonologists with ILD expertise, and radiologists with ILD expertise.

		**ALL**			**General Pulmonologists**			**General Radiologists**			**ILD Pulmonologists**			**ILD Radiologists**		
		Median	IQR		Median	IQR		Median	IQR		Median	IQR		Median	IQR	
**Definite UIP**	pre	0.64	0.61	0.66	0.64	0.62	0.67	0.64	0.61	0.67	0.63	0.60	0.66	0.63	0.60	0.65
	**post**	**0.67***	**0.64**	**0.69**	**0.67***	**0.65**	**0.69**	**0.67***	**0.64**	**0.69**	**0.67***	**0.63**	**0.69**	**0.65***	**0.63**	**0.68**
	change	0.03	0.01	0.04	0.02	0.01	0.04	0.02	0.00	0.04	0.03	0.02	0.04	0.03	0.01	0.04
**Probable UIP**	pre	0.60	0.57	0.64	0.61	0.57	0.65	0.60	0.57	0.64	0.59	0.57	0.63	0.59	0.56	0.62
	**post**	**0.65***	**0.63**	**0.68**	**0.66***	**0.63**	**0.69**	**0.65***	**0.63**	**0.68**	**0.66***	**0.63**	**0.68**	**0.65***	**0.63**	**0.66**
	change	0.05	0.02	0.07	0.04	0.01	0.07	0.04	0.02	0.06	0.05	0.03	0.08	0.05	0.02	0.07
**Indeterminate**	pre	0.61	0.57	0.65	0.62	0.59	0.66	0.60	0.55	0.63	0.61	0.57	0.65	0.61	0.57	0.64
	**post**	**0.63***	**0.60**	**0.65**	**0.63***	**0.61**	**0.66**	**0.62***	**0.57**	**0.64**	**0.62***	**0.59**	**0.65**	**0.62***	**0.59**	**0.65**
	change	0.01	-0.01	0.03	0.01	-0.01	0.02	0.01	0.00	0.03	0.01	-0.01	0.03	0.01	0.00	0.03
**Alternative diagnosis**	pre	0.55	0.51	0.58	0.55	0.52	0.58	0.54	0.51	0.59	0.54	0.51	0.57	0.54	0.51	0.56
	**post**	0.55	0.52	0.57	0.55	0.52	0.57	0.54	0.52	0.57	0.55	0.53	0.57	0.54	0.52	0.57
	change	0.00	-0.03	0.03	0.00	-0.05	0.03	-0.01	-0.04	0.02	0.01	-0.02	0.04	0.01	-0.01	0.03

Pre- to post- change in C-index was compared using a Wilcoxon signed rank test, and between group differences in change scores were compared using a Kruskal-Wallis test (4 groups) with a significance level of $p<0.05$. *Indicates a significant difference pre to post; †Indicates significant group differences in change scores.

Table S10. Change in concordance (c-index) between each reviewer's scores and 12-month disease progression before (pre) and after (post) viewing SOFIA outputs for radiologists and pulmonologists.

		Radiologists			Pulmonologists		
		Median	IQR		Median	IQR	
**Definite UIP**	pre	0.63	0.60	0.66	0.64	0.61	0.66
	**post**	**0.66***	**0.63**	**0.69**	**0.67***	**0.64**	**0.69**
	change	0.02	0.01	0.04	0.03	0.01	0.04
**Probable UIP**	pre	0.60	0.57	0.64	0.61	0.57	0.64
	**post**	**0.65***	**0.63**	**0.67**	**0.66***	**0.63**	**0.69**
	change	0.04	0.02	0.06	0.05	0.02	0.07
**Indeterminate**	pre	0.60	0.57	0.64	0.62	0.58	0.66
	**post**	**0.62***	**0.58**	**0.64**	**0.63***	**0.60**	**0.65**
	change	0.01	0.00	0.03	0.01	-0.01	0.03
**Alternative**	pre	0.54	0.51	0.57	0.55	0.52	0.58
**diagnosis**	**post**	0.54	0.52	0.57	0.55	0.52	0.57
	change	0.00	-0.03	0.03	0.00	-0.03	0.03

Pre- to post- change in C-index was compared using a Wilcoxon signed rank test, and between group differences in change scores were compared using a Mann-Whitney U test (2 groups) with a significance level of $p<0.05$. *Indicates a significant difference pre to post; †Indicates significant group differences in change scores.

Table S11. Change in concordance (c-index) between each reviewer's scores and 12-month disease progression before (pre) and after (post) viewing SOFIA outputs for reviewers with and without ILD expertise.

		Reviewers with ILD expertise			Reviewers without ILD expertise		
		Median	IQR		Median	IQR	
**Definite UIP**	pre	0.63	0.60	0.65	0.64	0.61	0.67
	**post**	**0.66***	**0.63**	**0.69**	**0.67***	**0.64**	**0.69**
	change	0.03	0.02	0.04	0.02	0.01	0.04
**Probable UIP**	pre	0.59	0.57	0.63	0.61	0.57	0.65
	**post**	**0.65***	**0.63**	**0.68**	**0.66***	**0.63**	**0.68**
	change	**0.05†**	**0.02**	**0.07**	**0.04**	**0.01**	**0.07**
**Indeterminate**	pre	0.61	0.57	0.65	0.62	0.58	0.65
	**post**	**0.62***	**0.59**	**0.65**	**0.63***	**0.60**	**0.65**
	change	0.01	0.00	0.03	0.01	-0.01	0.03
**Alternative**	pre	0.54	0.51	0.57	0.55	0.52	0.58
**diagnosis**	**post**	**0.55***	**0.53**	**0.57**	0.54	0.52	0.57
	change	**0.01†**	**-0.02**	**0.03**	**0.00**	**-0.04**	**0.02**

Pre- to post- change in C-index was compared using a Wilcoxon signed rank test, and between group differences in change scores were compared using a Mann-Whitney U test (2 groups) with a significance level of $p<0.05$. *Indicates a significant difference pre to post; †Indicates significant group differences in change scores.

Table S12. Change in concordance (c-index) between each reviewer's scores and 12-month disease progression before (pre) and after (post) viewing SOFIA outputs for reviewers with the greatest and least number of years of experience (top and bottom quartile, respectively).

		**Most experienced reviewers**			**Least experienced reviewers**		
		Median	IQR		Median	IQR	
**Definite UIP**	pre	0.63	0.61	0.66	0.64	0.62	0.67
	**post**	**0.67***	**0.63**	**0.69**	**0.67***	**0.65**	**0.70**
	change	0.02	0.01	0.04	0.02	0.01	0.04
**Probable UIP**	pre	0.61	0.57	0.63	0.61	0.59	0.65
	**post**	**0.65***	**0.62**	**0.68**	**0.66***	**0.64**	**0.69**
	change	0.04	0.02	0.07	0.05	0.02	0.07
**Indeterminate**	pre	0.61	0.58	0.64	0.61	0.58	0.65
	**post**	**0.63***	**0.59**	**0.65**	**0.63***	**0.60**	**0.65**
	change	0.01	-0.01	0.04	0.01	0.00	0.03
**Alternative diagnosis**	pre	0.55	0.52	0.57	0.54	0.51	0.57
	**post**	0.55	0.53	0.57	0.54	0.52	0.57
	change	0.01	-0.04	0.04	0.01	-0.02	0.04

Pre- to post- change in C-index was compared using a Wilcoxon signed rank test, and between group differences in change scores were compared using a Mann-Whitney U test (2 groups) with a significance level of $p<0.05$. *Indicates a significant difference pre to post; †Indicates significant group differences in change scores.

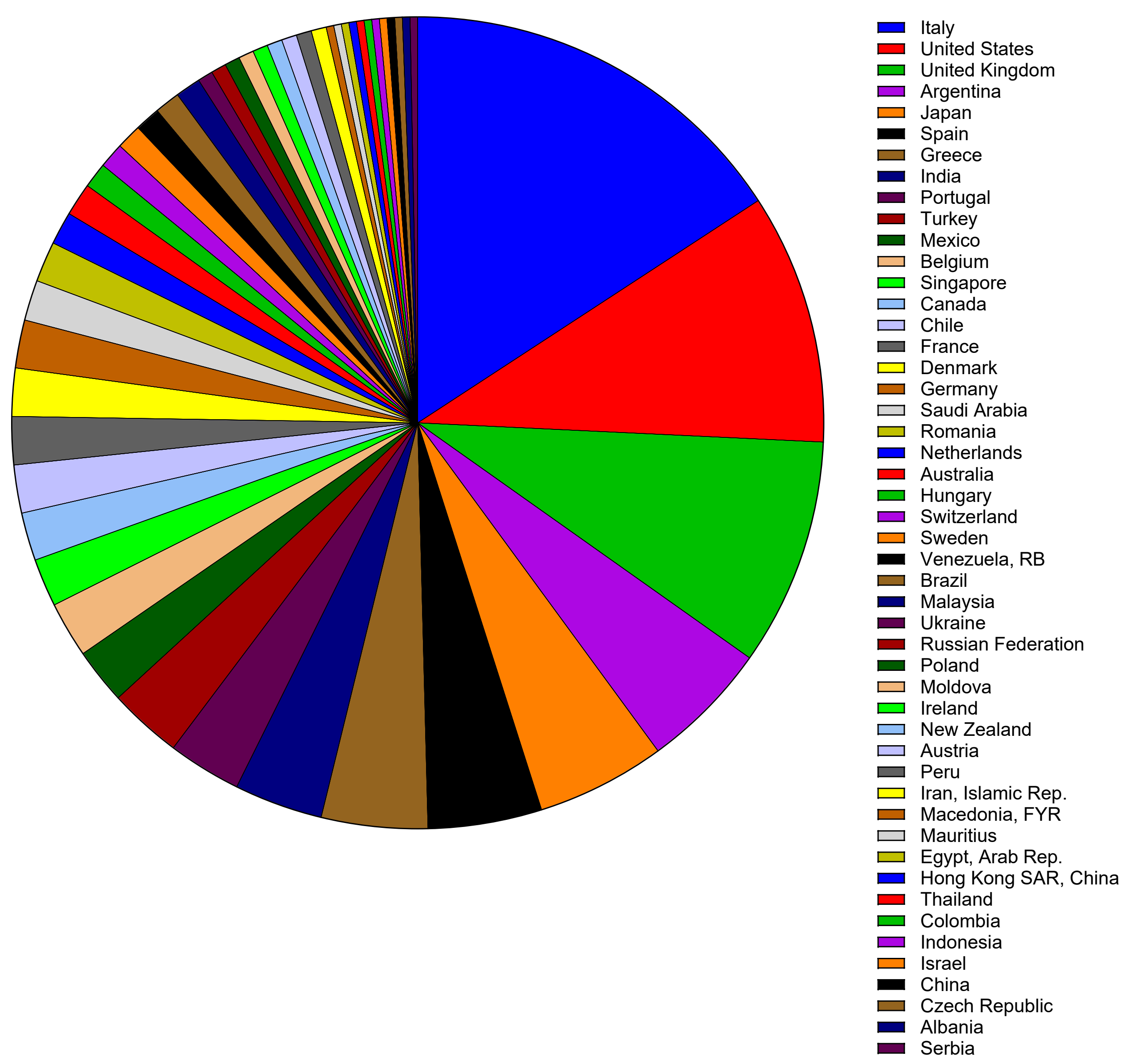

Figure S1. Distribution of reviewer locations by country.
