# Supplementary material for: Deep‐Learning Algorithm Diagnostic Support for Usual Interstitial Pneumonia Pattern Recognition in Fibrotic Interstitial Lung Disease
Source: Respirology. 2026 Apr 1;31(7):711–20. doi: 10.1002/resp.70246 (PMC13342441; doi:10.1002/resp.70246)
Supplement: Supplementary file 2 — Data S1: The SOFIA Project Consortium. [file RESP-31-711-s001.docx]

**Collaborators.**

The SOFIA Project Consortium:

Daniele Accornero, Aditya Agrawal, Isil Kibar Akilli, Omer Alamoudi, Maria Laura Alberti, Rasoul Aliannejad, Hamdan Aljahdali, Gina Amanda, Reut Anconina, Julio Daniel Antuni, Giuseppe Aquaro, Juan Arenas-Jimenez, Bassey Asuquo, Iain Au-Yong, Sergey Avdeev, Maurizio Balbi, Bruno Baldi, Elisabetta Balestro, Andrea Yu-Lin Ban, Fotini Bardaka, Nicola Boscolo Bariga, Dhiraj Baruah, Ionela Belaconi, Elisabeth Bendstrup, David Bennett, Hans-Christian Blum, Marialuisa Bocchino, Samuel De Bontridder, Andrea Borghesi, Demosthenes Bouros, Gracijela Bozovic, Pierre-Yves Brillet, Marsel Broqi, John Bruzzi, Suryakala Buddha, Ivette Buendia-Roldan, Carolina Cabo, Maria del Carmen Venero Caceres, Cristina Calandra, Roberto Calandriello, Diana Calaras, Jack Callum, Paula Campos, Roberto Carbone, Fabian Caro, Andre Carvalho, Marcelo Figueroa Casas, Eva Castaner, Jesus Javier Diaz Castanon, Cecilio Ceballos, Lorenzo Cereser, Veli Cetinsu, Gin Tsen Chai, Sachin Chaudhary, Nazia Chaudhuri, Chih-Yu Chen, Patrick Alain Chui Wan Cheong, Giuseppe Cicchetti, Annemilia del Ciello, Alessandro Balbiano di Colcavagno, Sahary Conde, Pietro Costantini, Vincent Cottin, Davide Coviello, Diletta Cozzi, Dumitru Cravcenco, Giuseppe Cutaia, Rosa D’Abronzo, Gabriele D’Andrea, Marie-Pierre Debray, Perla Delgado, Diemen Delgado-Garcia, Alain Delobbe, Jane Dematte, Devesh J Dhasmana, Sahajal Dhooria, Fotios Drakopanagiotakis, Dildar Duman, Chary Duraikannu, Glenn Eiger, Karim El-Kersh, Samantha Ellis, Juan Ignacio Enghelmayer, Rosa Estrada-Y-Martin, Sherene Fakhran, Alessandra Farchione, Mary Jo Farmer, Alexia Farrugia, Leandro Fassola, Paola Faverio, Federico Felder, Maria Fernandez-Velilla, Gilbert Ferretti, Justyna Fijolek, Francesco Filippone, Kevin Flaherty, Fabio Franco, Domenico Salvatore Gagliano, Amaia Urrutia Gajate, Clara Patricia Garcia, Andrea Estrada Garrido, Adrian Gaser, Bindu George, Subha Ghosh, Eddie Gibson, Hester Gietema, Rodrigo Gil, Beatriz Liliana Gil, Ritu Gill, Georgia Gkrepi, Raul Godoy, Athena Gogali, Nicole Goh, Alejandro Gomez, Aleksandar Grgic, Julien Guiot, H Henry Guo, Amit Gupta, Richard Hammond, Simon Hart, Thomas Hartman, Michael Henry, Nik Hirani, Wan Chin Hsieh, Killian Hurley, Charlotte Hyldgaard, Daniela Buklioska Ilievska, Marta Inchausti, Yoshikazu Inoue, Dominique Israel-Biet, Maham Jehangir, Kerri Johannson, Takeshi Johkoh, Janet Johnston, Fortunato Juarez-Hernandez, Soma Jyothula, Yasemin Kabasakal, Meena Kalluri, Can Zafer Karaman, Peter Kardos, Sandeep Katiyar, Ravindra Kumar Kedia, Lan-Chau Kha, Nasreen Khalil, Mohammad Ayaz Khan, Yet H Khor, Arda Kiani, Tomoo Kishaba, Heiko Knoop, Umut Knoop, Jane Ko, Eva Kocova, Lykourgos Kolilekas, Yasuhiro Kondoh, Chi Wan Koo, Vasileios Kouranos, Martijn de Kruif, Melahat Kul, Ozlem Ozdemir Kumbasar, Ronald Kuzo, Hoi Yee Kwan, Sebastien Van Laethem, Nicholas Landini, David Lang, Anna Rita Larici, Esther Law, Roberta Eufrasia Ledda, Ivo van der Lee, Yunkai Li, Valencia Lim, Randolph Lipchik, Su Ying Low, Fabrizio Luppi, Foteini Malli, Milena Adina Man, Eliane Mancuzo, Silvina Mannarino, George Margaritopoulos, Cristina Marrocchio, Manuela Martinez-Frances, Toshiaki Matsuda, Federico Mei, Mayra Mejia, Veronica Menardi, Mikel Mendoza, Aravind Menon, Patricia Lopez Miguel, Ruxandra-Iulia Milos, Paul Minnis, Atsushi Miyamoto, Nesrin Mogulkoc, Maria Molina-Molina, Michele Mondoni, Zsuzsanna Monostori, Brian Morrissey, Marta Garcia Moyano, Mathias Andreas Mueller, Suranjan Mukherjee, Carlos F Munoz-Nunez, Daniel Musetescu, Prasanth Nair, Anoop Nambiar, Kiran Vishnu Narayan, Hrudaya Nath, Yuichiro Nei, Alexandra Neves, Boon Hau Ng, Tilo Niemann, Luca Novelli, Lubov Novikova, Paschalis Ntolios, Hilario Nunes, Takashi Ogura, Shinichiro Ohshimo, Anastasia Oikonomou, Maria Otaola, Marieke Overbeek, Lekshmi Padmakumari, Stefano Palmucci, Vijaya Kumary Baskara Pandian, Eftsratios Panselinas, Ilias Papanikolaou, Alessio Pascheale, Shital Patil, Wagner Diniz de Paula, Michael Perch, Raoul Pereira, Olof Joakim Pettersson, Sara Piciucchi, Wojciech Piotrowski, Roberta Polverosi, Daniel Popa, Ana Sofia Porta, Marta Posada, Thomas Skovhus Prior, Ilaria Pulzato, Mosleh Al Raddadi, Pailin Ratanawatkul, Gaetano Rea, Cristina Reichner, Pilar Rivera-Ortega, Jonathan Rodrigues, Rui Rolo, Shigeki Saito, Yoana Lazaro Salazar, Mauricio Salinas, Mayra Alexandra Samudio, Pradosh Kumar Sarangi, Sayan Sarkar, Yuki Sato, Recep Savas, Simone Scarlata, Nicola Schembri, Thies Hendrik Schroeder, Alfredo Sebastiani, Palmi Shah, Aruna Shanmuganathan, Claudio Silva, Hans Slabbynck, Philip Slocum, Annemiek Snoeckx, Eman Sobh, Chun Ian Soo, Celia Sousa, Mark Spears, Irina Strambu, Emilia Maria Swietlik, Anne-Marie Sykes, Pablo Szwarstein, Gabriela Tabaj, Yoshinori Tanino, Kiminobu Tanizawa, Adam Domonkos Tarnoki, David Laszlo Tarnoki, Felicia Teo, Weiping Tham, Fernando Tirapegui, Ryuichi Togawa, Claudia Lucia Toma, Sara Tomassetti, Keisuke Tomii, Hiromi Tomioka, Ioannis Tomos, Abdelfattah Touman, Sergio Trujillo, Vasilios Tzilas, Argyris Tzouvelekis, Marcela Usandivaras, Clara Valsecchi, Francesco Varone, Gerson Velasquez-Pinto, Oksana Viltsaniuk, Yagnang Vyas, Yuko Waseda, Yuranga Weerakkody, Margaret Wilsher, Wim Wuyts, Oleh Yakovenko, Esteban Zirulnik, Maurizio Zompatori
